# Supplementary material for: Multi-gene incongruence consistent with hybridisation in Cladocopium (Symbiodiniaceae), an ecologically important genus of coral reef symbionts
Source: PeerJ. 2019 Jun 25;7:e7178. doi: 10.7717/peerj.7178 (PMC6598746; doi:10.7717/peerj.7178)
Supplement: Supplemental Information 2 — Two figures, one table and one methods section providing additional information. [file peerj-07-7178-s002.docx]

**Multi-gene incongruence consistent with hybridisation in *Cladocopium* (Symbiodiniaceae), an ecologically important genus of coral reef symbionts: Supplementary Information**

**Authors**

Joshua I. Brian, Simon K. Davy, Shaun P. Wilkinson


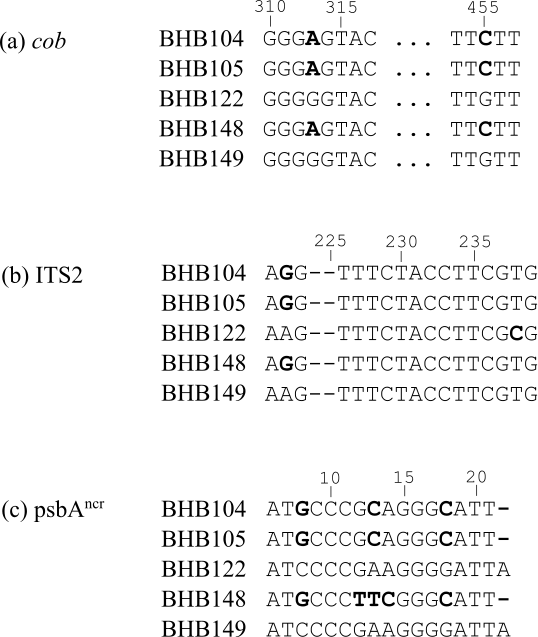


Figure S1: Short selections of raw sequence data for non-incongruent sample BHB148 and related sequences (polymorphisms in bold). For all three gene regions (a) – (c), BHB148 groups with the samples BHB104 and BHB105. It is likely the reticulate pattern observed for this sample in Fig. 3b and Fig. 3c is caused by additional unique variation in base pairs 12-14 in the psbA^ncr^ region, which has led to BHB148 being designated as sister to the wrong subgroup in psbA^ncr^ trees.


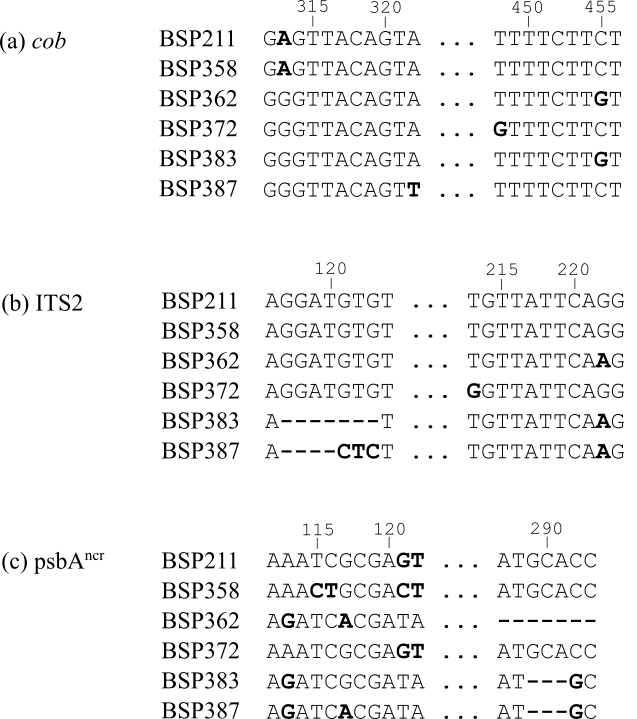


Figure S2: Short selections of raw sequence data for non-incongruent samples BSP358, BSP72, BSP387 and related sequences (polymorphisms in bold). There is significant variation in these sequences, however in general it is clear there are two distinct groups recovered by the ITS2 and psbA^ncr^ regions: {BSP211, BSP358, BSP372} and {BSP362, BSP383, BSP387}. This is less clear in the *cob* gene, thanks to point mutations at bases 448 and 322 for samples BSP372 and BSP387 respectively, in addition to two unique point mutations in sample BSP358 at base pairs 154 and 163 (not presented here). This additional variation in the highly homogeneous *cob* gene explains the incongruences observed in Fig. 4a and 4c, whereas the raw sequences are consistent with a linear evolutionary history in these samples.

Table S1: Evolutionary models selected by PAUP* for each dataset for building maximum likelihood trees, for the AU tests. HKY = Hasegawa, Kishino and Yano (1985); GTR = Generalised Time Reversible (Tavaré 1986); F81 = Felsenstein (1981); JC = Jukes and Cantor (1969); K80 = Kimura (1980); K81 = Kimura (1981); SYM = Symmetrical (Zharkikh 1994). G = gamma coefficient, I = proportion of invariant sites.

| **Dataset** | **Gene Region** | **Model** | **I** | **G** |
| --- | --- | --- | --- | --- |
| BBR | *cob* | GTR+I | 0.921 | - |
|  | ITS2 | K81+I | 0.654 | - |
|  | psbA^ncr^ | SYM+G | - | 0.548 |
| BHB | *cob* | GTR+I | 0.918 | - |
|  | ITS2 | HKY | - | - |
|  | psbA^ncr^ | SYM+G | - | 0.502 |
| BLS | *cob* | GTR+I | 0.924 | - |
|  | ITS2 | K81+I | 0.648 | - |
|  | psbA^ncr^ | JC+G | - | 0.693 |
| BSP | *cob* | GTR | 0.833 | - |
|  | ITS2 | K80 | - | - |
|  | psbA^ncr^ | SYM+G | - | 0.528 |
| HEW | *cob* | GTR+I | 0.850 | - |
|  | ITS2 | K80 | - | - |
|  | psbA^ncr^ | SYM+G | - | 0.606 |
| LIE | *cob* | GTR+I | 0.921 | - |
|  | ITS2 | K80 | - | - |
|  | psbA^ncr^ | SYM+G | - | 0.706 |
| LIW | *cob* | GTR | - | - |
|  | ITS2 | K80 | - | - |
|  | psbA^ncr^ | SYM+G | - | 0.526 |
| Atauro | *cob* | F81+I | 0.875 | - |
|  | ITS2 | K80 | - | - |
|  | psbA^ncr^ | SYM+G | - | 0.460 |
| Timor | *cob* | GTR | - | - |
|  | ITS2 | K80 | - | - |
|  | psbA^ncr^ | K80+G | - | 0.503 |

**Supplementary Methods: DNA Amplifications**

*Direct Sequencing*

For all *cob*, psbA^ncr^ and host ITS2 amplifications, PCR reactions consisted of 1 $\times$ MyTaq HS Red Mix (BioLine, Randolph, MA, USA), ~20 ng sample DNA, 10 µg BSA, 0.25 µM of each primer and H_2_O to a total volume of 20 µL, and used a Veriti 96-well thermal cycler (Applied Biosystems). Amplicons were purified with MagNA solution (0.1% carboxyl-modified Sera-Mag Magnetic Speed-Beads (Fisher Scientific), 18% w/v PEG-8000, 1M NaCl, 10mM Tris-HCl, 1 mM EDTA, 0.05% Tween 20, pH 8.0; Rohland and Reich 2012).

The *cob* gene amplifications with the pair Dinocob1F/Dinocob1R (Zhang *et al.* 2005) had cycling conditions of 1 min denaturation at 95ºC, followed by 40 cycles of 15 s at 95ºC, 20 s at 55ºC, 30 s at 72ºC, and a final extension for 7 min at 72ºC. *cob* gene amplifications with the pair Cob_f1/Cob_r1 (Pochon *et al*. 2012) had cycling conditions of 2 min denaturation at 95ºC, followed by 40 cycles of 20 s at 95ºC, 20 s at 58ºC, 30 s at 72ºC, and a final extension for 7 min at 72ºC. *Cladocopium* psbA^ncr^ amplifications with the primer pair 7.4-Forw/7.8-Rev (Moore *et al*. 2003) had the cycling conditions of 3 min denaturation at 95ºC, followed by 38 cycles of 20 s at 95ºC, 30 s at 57ºC, 30 s at 72ºC, and a final extension for 7 min at 72ºC.

*Next Generation Sequencing*

The primary amplification using the primers ITSD (Pochon *et al.* 2001)/ITS2Rev2 (Stat *et al*. 2009) with Illumina adapters attached consisted of 1 $\times$ SuperFi High-Fidelity MasterMix (Invitrogen), 10 ng sample DNA, 10 µg BSA, 0.2 µM of each primer and H_2_O to 25 µL. Cycling conditions were a denaturation for 3 min at 95ºC, followed by 25 cycles of 30 s at 95ºC, 30 s at 55ºC, 30 s at 72ºC, and a final extension for 5 min at 72ºC. Amplicons were purified using MagNA solution as above, and the second indexing PCR run of 8 cycles (same conditions) used 1 $\times$ SuperFi MasterMix, 2 µL of PCR product from the initial amplification, 0.1 µM each of a unique forward/reverse primer combination, and H_2_O to 20 µL.

qPCR reactions consisted of 1 $\times$ SYBR Green Mix (BioLine), 0.5 µM of each of the primers ITSD/ITS2Rev2, 20 µg BSA, 2 µL of the indexed samples (diluted 1:1000) and H_2_O to 10 µL. Cycling conditions were a denaturation for 10 min at 95ºC, followed by 40 cycles of 15 s at 95ºC and 1 min at 60ºC. Samples were run in duplicate. C_T_ (cycle threshold) values were generated *via* a calculation (using an in-built machine algorithm) of when the DNA signal differed significantly from background fluorescence, using a baseline of 0.1. A melt curve was run (temperature elevation from 60°C to 95°C in 0.3°C increments each of 15 s duration) to ensure that only the target sequences were amplified. Samples were considered successful if duplicates had a standard deviation in C_T_ values <0.5 and melted within 1ºC of the average melting temperature of the target region (~82ºC); samples that failed these were re-done. DNA concentrations were calculated from C_T_­ values using a standard curve which was run using previously prepared clonal serial dilutions of known concentration (Wilkinson *et al.* 2015), and different volumes of the final purified samples were pooled in a single tube to achieve equal concentration of all samples, with the concentration of the final pooled library being 4 nM DNA.

**Supplementary Information References**

Felsenstein J (1981) Evolutionary trees from DNA sequences: a maximum likelihood approach. *Journal of molecular evolution*, **17***,* 368-376.

Hasegawa M, Kishino H, Yano TA (1985) Dating of the human-ape splitting by a molecular clock of mitochondrial DNA. *Journal of molecular evolution*, **22**, 160-174.

Jukes TH, Cantor CR (1969) Evolution of protein molecules. *Mammalian protein metabolism*, **3***,* 132.

Kimura M (1980) A simple method for estimating evolutionary rates of base substitutions through comparative studies of nucleotide sequences. *Journal of molecular evolution*, **16**, 111-120.

Kimura M (1981) Estimation of evolutionary distances between homologous nucleotide sequences. *Proceedings of the National Academy of Sciences*, **78**, 454-458.

Moore RB, Ferguson KM, Loh WK, Hoegh-Guldberg O, Carter DA (2003) Highly organized structure in the non-coding region of the psbA minicircle from clade C *Symbiodinium*. *International Journal of Systematics and Evolutionary Microbiology*, **53**, 1725-1734.

Pochon X, Pawlowski J, Zaninetti L, Rowan R (2001) High genetic diversity and relative specificity among *Symbiodinium*-like endosymbiotic dinoflagellates in soritid foraminiferans. *Marine Biology*, **139**, 1069-1078.

Pochon X, Putnam HM, Burki F, Gates RD (2012) Identifying and characterizing alternative molecular markers for the symbiotic and free-living dinoflagellate genus *Symbiodinium*. *PLoS One*, **7**, e29816.

Rohland N, Reich D (2012) Cost-effective, high-throughput DNA sequencing libraries for multiplexed target capture. *Genome Research*, **22**, 939-946.

Stat M, Pochon X, Cowie RO, Gates RD (2009) Specificity in communities of Symbiodinium in corals from Johnston Atoll. *Marine Ecology Progress Series*, **386**, 83-96.

Tavaré S (1986) Some probabilistic and statistical problems in the analysis of DNA sequences. *Lectures on mathematics in the life sciences*, **17**, 57-86.

Wilkinson SP, Fisher PL, van Oppen MJH, Davy SK (2015) Intra-genomic variation in symbiotic dinoflagellates: recent divergence or recombination between lineages?, *BMC Evolutionary Biology*, **15**, 46

Zhang H, Bhattacharya D, Lin S (2005) Phylogeny of dinoflagellates based on mitochondrial cytochrome B and nuclear small subunit rDNA sequence comparisons. *Journal of Phycology,* **41**, 411-420.

Zharkikh A (1994) Estimation of evolutionary distances between nucleotide sequences. *Journal of molecular evolution*, **39**, 315-329.
